# Rice OsLHT1 Functions in Leaf-to-Panicle Nitrogen Allocation for Grain Yield and Quality

**DOI:** 10.3389/fpls.2020.01150

**Published:** 2020-07-29

**Authors:** Nan Guo, Mingji Gu, Jinqi Hu, Hongye Qu, Guohua Xu

**Affiliations:** State Key Laboratory of Crop Genetics and Germplasm Enhancement, MOA Key Laboratory of Plant Nutrition and Fertilization in Lower-Middle Reaches of the Yangtze River, Nanjing Agricultural University, Nanjing, China

**Keywords:** rice, amino acids, transporter, OsLHT1, nitrogen allocation, grain yield, grain quality

## Abstract

Proper allocation of nitrogen (N) from source leaves to grains is essential step for high crop grain yield and N use efficiency. In rice (*Oryza sativa*) grown in flooding paddy field, amino acids are the major N compounds for N distribution and re-allocation. We have recently identified that *Lysine-Histidine-type Transporter 1* (*OsLHT1*) is the major transporter for root uptake and root-to-shoot allocation of amino acids in rice. In this study, we planted knockout mutant lines of *OsLHT1* together wild-type (WT) in paddy field for evaluating OsLHT1 function in N redistribution and grain production. OsLHT1 is expressed in vascular bundles of leaves, rachis, and flowering organs. *Oslht1* plants showed lower panicle length and seed setting rate, especially lower grain number per panicle and total grain weight. The concentrations of both total N and free amino acids in the flag leaf were similar at anthesis between *Oslht1* lines and WT while significantly higher in the mutants than WT at maturation. The *Oslht1* seeds contained higher proteins and most of the essential free amino acids, similar total starch but less amylose with lower paste viscosity than WT seeds. The mutant seeds showed lower germination rate than WT. Knockout of *OsLHT1* decreased N uptake efficiency and physiological utilization efficiency (kg-grains/kg-N) by about 55% and 72%, respectively. Taken together, we conclude that OsLHT1 plays critical role in the translocation of amino acids from vegetative to reproductive organs for grain yield and quality of nutrition and functionality.

## Introduction

In natural soil ecosystem, nitrogen (N) is presented in diversity forms including both inorganic and organic N (Rasmussen et al., 2010; Robertson and Groffman, 2015). Rice (*Oryza sativa*) is commonly grown in flooding paddy filed where ammonium is dominant form of inorganic N (Xu et al., 2012). Rice absorbs N rapidly at the vegetative stage, especially at the early stage of growth (Muhammad and Kumazawa, 1974; Masclaux-Daubresse et al., 2010). When the panicles emerge, the N is highly distributed to both the flag leaves and panicles (Muhammad and Kumazawa, 1974; Itoh et al., 2005). During grain-filling stage, the N supplied for panicles is mainly redistributed from leaves and about 70% of N can be transported into the panicle (Muhammad and Kumazawa, 1974). The leaf blades are the main organ of photosynthesis and the N in the flag leaf contributes significantly to the yield (Ma et al., 1989).

In flooding paddy soil, ammonium absorbed in rice roots is further assimilated in plastid into amino acids which are then transferred from roots to leaves *via* xylem. Amino acids in leaves are loaded into sieve elements and companion cells complexes and released to seeds (Tegeder, 2012; Tegeder, 2014; Tegeder and Masclaux-Daubresse, 2018). Import of amino acids into phloem and seeds is the key event in source to sink partitioning of N, and the sink development and grain yield rely on the amounts of amino acids translocation (Santiago and Tegeder, 2016; Tegeder and Hammes, 2018). Several membrane proteins for amino acid allocation have been characterized recently and manipulation of these transporters is able to regulate plant growth. In *Arabidopsis*, AtLHT1, with the strong expression in the surface of roots, young leaves, flowers, and siliques, is involved in amino acid uptake and import into the mesophyll cells (Chen and Bush, 1997; Hirner et al., 2006). Disruption of *AtLHT1* exhibited decrease of shoot biomass and seed yield while *AtLHT1* overexpression improved the N use efficiency under limiting N (Hirner et al., 2006). Furthermore, AtAAP2 is expressed in the phloem and functions in xylem-to-phloem transfer step. Increased amino acids allocation to leaves in *aap2* mutants resulted in higher seed yields (Zhang et al., 2010; Perchlik and Tegeder, 2018). AtAAP8 is shown to be expressed in leaf phloem and siliques. It functions in amino acid loading into the phloem and import into seeds. Decreased amino acid loading into phloem and partitioning to sinks in *aap8* mutants led to decreased siliques and seed numbers (Schmidt et al., 2007; Santiago and Tegeder, 2016). AtAAP1 is localized to *Arabidopsis* embryos and regulates amino acid distribution to the developing embryos. Amino acid loaded into embryo *via AtAAP1* could also affect seed protein reserves and seed yield (Sanders et al., 2009). Similarly, in pea plants, overexpression of *Amino Acid Permease1* (*AAP1*) in the phloem and embryos was able to improve seed yield and N use efficiency (Zhang et al., 2015; Perchlik and Tegeder, 2017).

In rice, 85 putative amino acid transporters were identified (Zhao et al., 2012). However, only a few of them have been associated with N translocation from source leaves to grains. *OsAAP3* is localized to roots and leaves. Disruption of *OsAAP3* decreased amino acid levels in various tissues, and promoted the tiller growth, and further improved the grain yield (Lu et al., 2018). *OsAAP5* is expressed in leaves and panicles and regulates tiller growth by affecting cytokinin levels. Loss-of-function of *OsAAP5* could improve grain yield (Wang J. et al., 2019). Further, storage proteins act as a N sink and N allocation affects the composition of grain storage compounds (Perez et al., 1996; Chen et al., 2012). The major organic compounds of rice grains are starch, protein and lipids (Fitzgerald et al., 2009; Li et al., 2018). Amylose and amylopectin are the two types of starch (Shewry et al., 1995; Fitzgerald et al., 2009; Chen et al., 2012). Notably, OsAAP6 was revealed to function in affecting amino acid allocation and regulating grain protein content in rice (Peng B. et al., 2014).

Recently, based on the genetic association analysis between root ^15^N-aspartate uptake rate of rice core accessions and single-nucleotide polymorphisms of putative amino acids transporter genes, we found that *Lysine-Histidine-type Transporter 1 (OsLHT1)* is a key transporter for root amino acids uptake in rice (Guo et al., 2020). Repression of *OsLHT1* also decreased amino acids allocation from root to shoot and rice growth (Wang X. et al., 2019; Guo et al., 2020). Notably, the reduction in shoot biomass of *Oslht1* versus wild-type (WT) plants was accelerated during development (Guo et al., 2020). In this study, we analyzed the effect of *OsLHT1* knockout on N allocation from source leaves to developing grains and the components of storage proteins and starches in the grains. The results show that OsLHT1 contributes to forming grain yield and quality, and high N use efficiency in rice.

## Materials and Methods

### Plant Materials, Growth Conditions, and Harvest

Field experiments were conducted at the research base of Nanjing Agricultural University in Ledong county, Hainan Province, China. Seeds of *Oslht1* mutant lines with *Nipponbare* background (Guo et al., 2020) were germinated and planted according to Chen et al. (2016) and Fan et al. (2016). Fields were managed according to local agricultural practices and 180-kg N/ha was applied during the growth season. For molecular and physiological analysis, rice plants were harvested at both anthesis and maturity. Panicles, leaves, and culms were collected, dried at 60°C for 3 days and weighed. Panicles were considered as reproductive tissues while leaves and culms were considered as vegetative tissues.

### RNA Extraction, and Gene Expression Analysis

WT plants were planted in the paddy field (see above). At the anthesis stage, spikelet, rachis, leaf blades, leaf sheaths, nodes, and internodes were collected. Total RNA isolation and first-strand cDNA synthesis were performed according to Guo et al. (2020). RT-qPCR was performed with SYBR Premix Ex Taq™ II (Perfect Real Time) Kit (TaKaRa Biotechnology, Dalian, China) on the QuantStudio 6 Flex Real-Time PCR System (Applied Biosystems, MA, USA) using *OsLHT1*-specific primers (F: 5′;-GGACTCCGGCAGATCATCA; R: 5′;-CTGGTTTCATCATGTGTGCCTA). Relative expression levels were normalized to the rice housekeeping gene *OsActin1* using specific primer pairs (F: 5′;-CAACACCCCTGCTATGTACG; R: 5′;-CATCACCAGAGTCCAACACAA) and presented as 2^-△CT^.

### Analysis of OsLHT1 Promoter-GUS Line

Seeds of OsLHT1 promoter-GUS lines (Guo et al., 2020) were grown in the field (see above). GUS assays were done as described previously (Ai et al., 2009).

### Free Amino Acid Analysis

*Oslht1* mutant and WT plants were grown in the field (see above). At the anthesis or/and maturity stage, flag leaves and dry seeds were collected for amino acid analyses. Flag leaf free amino acid extraction was performed according to Peng B. et al. (2014). Amino acid contents in dry seeds were analyzed according to Zhou et al. (2009) and Wang et al. (2008) with some modifications. Rice grains were husked and ground to a flour consistency in a mill (JFS-13A, Hangzhou, China). Seed flour (0.100 g) was used for extraction with 5 ml of 6 M HCl in a sealed air-evacuated tube at 110°C for 22 hours. The hydrolysate was diluted to 100 ml. 1 ml dilution was dried in a Termovap Sample Concentrator MD200-1 (Allsheng, China) and re-dissolved in 1 ml water. Amino acid concentrations were determined using an L-8900 automatic amino acid analyzer (Hitachi, Tokyo, Japan). All steps were performed according to the manufacturer’s instructions. Most individual proteinous amino acid were analyzed, and results show the combined total amounts of Asn and Asp, and Gln and Glu, respectively (Peng B. et al., 2014; Guo et al., 2020).

### Analysis of Total N, Protein, Starch, Amylose Concentrations and Pasting Properties

*Oslht1* mutant and WT plants were grown in the field (see above). At the anthesis or/and maturity stage, panicles, leaves, culms, and dry seeds were collected for total elemental N, protein, starch, and amylose analyses. Rice tissues were husked and ground to a flour consistency in a mill (JFS-13A, Hangzhou, China). Total N content was analyzed according to Kjeldahl method (Chen et al., 2016). Protein content was obtained by multiplying 5.95 as the protein conversion factor (GB5009.5-2016, National Food Safety Standards, China). Total starch was extracted using a starch assay kit (Megazyme, Wicklow, Ireland) according to the manufacturer’s instructions and determined with ultraviolet spectrophotometer U-1800 (Hitachi, Tokyo, Japan). Amylose content was analyzed according to Liu et al. (2009). Rapid Visco Analyser (RVA, Perton, Warriewood, NSW, Australia) were used for evaluating pasting properties of endosperm of both *Oslht1* and WT seeds as described by Peng C. et al. (2014).

### Analysis of N Uptake Efficiency (NUpE) and N Utilization Efficiency (NUtE)

NUpE and NUtE were calculated according to Moll et al. (1982) and Xu et al. (2012). NUpE is the percentage ratio of total accumulated N in the above-ground shoot to total supplied fertilizer N. NUtE is the ratio of total grain yield to total accumulated N in the above-ground shoot.

### Seed Germination Analysis

A total of 100 respective fully filled seeds of *Oslht1* mutant lines and *Nipponbare* WT were surface-sterilized for 30 min in 30% (v/v) NaClO solution, washed with sterilized water and then placed in water in Petri dishes (d=15 cm) with two sheets of filter paper. The seeds were incubated at 28°C in the dark for 2 days and then moved into a tissue culture chamber with 14-hour-days at a light intensity of 6000 lux, 40% humidity, and temperatures of 28°C. The water was exchanged every day. Seeds were photographed and plant root length, shoot length and germination rate were measured.

### Statistical Analysis

Data are generally presented as means ± SD (standard deviation) of at least 5 biological repetitions. Significant differences were analyzed using IBM SPSS Statistics 20 program at one-way ANOVA followed by Tukey’s test.

### Accession Numbers

Sequence data from this article can be obtained from The Rice Genome Annotation Project (http://rice.plantbiology.msu.edu/) under the following accession numbers: *Oryza sativa japonica* LOC_Os08g03350 (*OsLHT1*), LOC_Os03g50885 (*OsActin1*).

## Results

### *OsLHT1* Is Highly Expressed in Vascular Bundles of Leaves and Rachis

**Figure 1:**
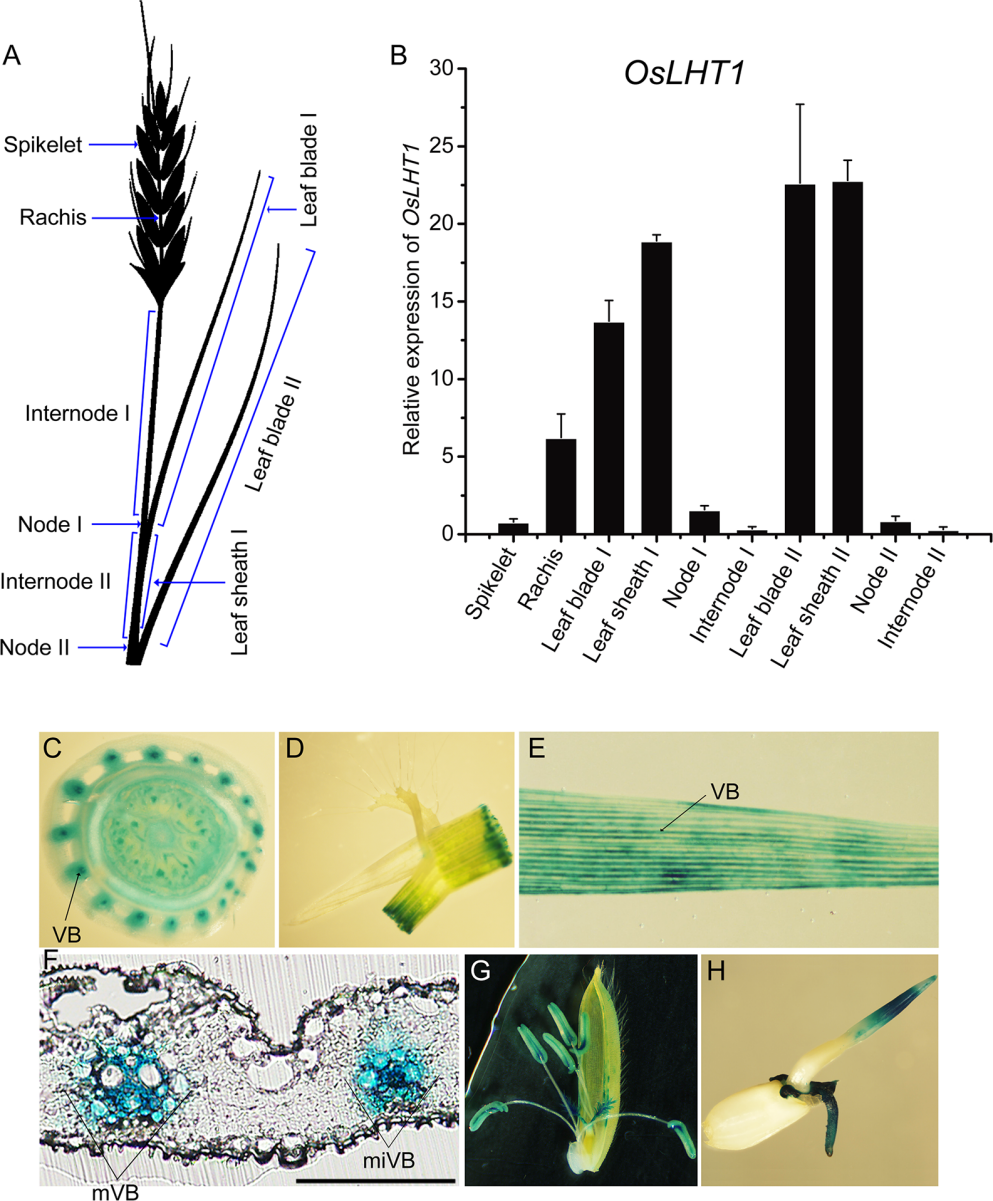
Expression levels of *OsLHT1* in rice. Rice (cv. *Nipponbare*) was grown in paddy field (see materials and methods). **(A)** The individual organ of the plant at the anthesis stage to be sampled for extraction of total RNA. **(B)** The relative expression of *OsLHT1* quantified by RT-qRCR. Rice housekeeping gene OsActin1 was used as an internal control. Values are means ± SD (n=5 biological replicates). **(C–E)** GUS staining in root-shoot junction **(C)**, leaf sheath-leaf blade junction **(D)**, and vascular bundles (VB) of mature leaf blades **(E)**. **(F)** Cross-sections of a leaf blade showing a major (mVB) and minor (miVB) vascular bundles. Scale bar: 100 μm. **(G)** GUS staining in flower and lemma. **(H)** GUS staining in three-day-old germinated seeds.

We have previously shown that *OsLHT1* transcripts were detected in roots and leaf blades at the seedling stage (Guo et al., 2020). Here we show *OsLHT1* expression highly in leaf blades, leaf sheaths and rachis, and relatively weak in spikelet, node and internode at the anthesis stage (**Figures 1A, B**). Since the leaf I (the youngest or flag leaf) and developing panicles are growing organs and need large amount of nitrogenous compounds transported *via* stem from leaves (Muhammad and Kumazawa, 1974), the abundant expression of *OsLHT1* in leaves and rachis hints that OsLHT1 may play a role in leaf-to-panicle amino acid allocation. GUS staining was observed throughout all cell types of root-shoot junction, with the strong expression levels in the vascular bundles (**Figure 1C**). GUS activity was present in leaf sheaths and leaf blades, specifically in the cells of the major and minor veins (**Figures 1D–F**). In addition, *OsLHT1* is strongly expressed in flowering organs, hulls and germinated seeds (**Figures 1G, H**), demonstrating its potential role in rice reproductive growth.

### Knockout of *OsLHT1* Suppressed Development of Both Panicles and Grains

**Figure 2:**
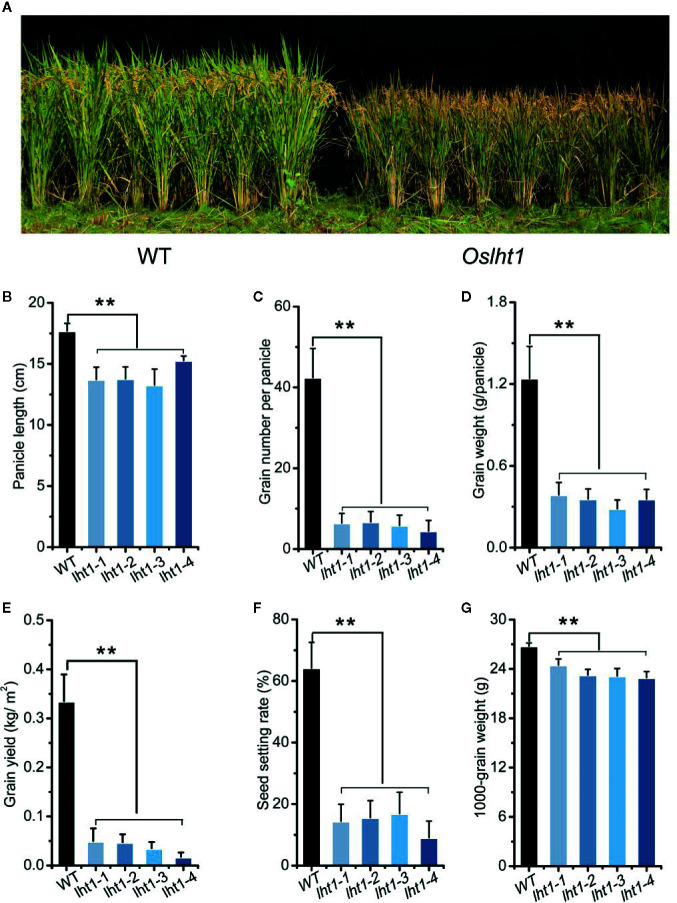
Effect of *OsLHT1* mutation on rice growth and grain yield components. Both wild-type (WT, cv. *Nipponbare*) and *Oslht1* mutants were grown in paddy field and harvested at the maturity stage for determining yield components (see *Materials and Methods*). **(A)** Image of *Oslht1* and WT plants in the field. **(B)** Mean panicle length. **(C)** Total grain number per panicle. **(D)** Total grain weight per panicle. **(E)** Total grain yield per plant. (F) Mean seed setting rate. **(G)** Mean 1000-seeds weight. Values are means ± SD (n = 7). Asterisks indicate significant differences between each *Oslht1* mutant line and WT detected by one-way ANOVA followed by Tukey’s test. **p < 0.01.

To detect the contribution of OsLHT1 in rice grain production, we compared the performance of WT and *Oslht1* mutants in the paddy field. At the maturation, in comparison to WT, *Oslht1* plants showed shorter height, brown leaf and spikelet (**Figure 2A**), shorter panicle length (**Figure 2B**), lower grain number per panicle, seed setting rate, grain weight per panicle and total grain yield per plant (**Figures 2C–F**). The *OsLHT1-*mutation reduced 1000-grain weight about 10% (**Figure 2G**), which was relatively less affected in comparison to the other parameters.

### Knockout of *OsLHT1* Affected Distribution of Different N Components in Leaf and Grains

**Figure 3:**
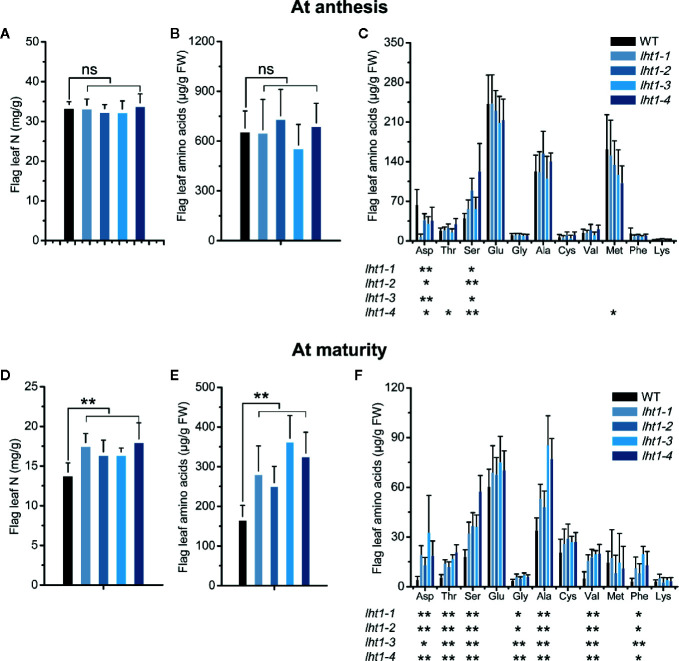
Effect of *OsLHT1* mutation on content of total N, total, and individual free amino acid in rice leaf at anthesis and maturity. Both wild-type (WT, cv. *Nipponbare*) and *Oslht1* mutants were grown in paddy field until mature. The flag leaf (fully expanded youngest leaf from top) of WT and *Oslht1* plants was sampled for the analysis of total N **(A, D)**, total free amino acids **(B, E)**, and individual free amino acid **(C, F)**. FW, fresh weight. Values are means ± SD (n = 7). Asterisks indicate significant differences between each *Oslht1* mutant line and WT detected by one-way ANOVA followed by Tukey’s test. *p < 0.05; **p < 0.01; ns, not significant.

In cereal crops, there is a considerably higher distribution of N from flag leaf to the panicle after the emergence of panicles (Muhammad and Kumazawa, 1974; Zhang et al., 2007; Chen et al., 2016; Tegeder and Masclaux-Daubresse, 2018). As expected, the contents of both total N and free amino acids in the flag leaf was dramatically decreased from anthesis to mature stage in both WT and *Oslht1* mutant lines of rice (**Figures 3A, D**), confirming that large portion of grain N was allocated from the flag leaf in addition of direct contribution of root acquired N from soil. Remarkably, there was no significant difference of the content of total N (mg/g), total free amino acids and most of individual free amino acids (mg/kg FW) in the flag leaf between WT and *Oslht1* plants at the anthesis stage (**Figures 3A–C**). However, their contents were significantly higher except similar level of aspartate, asparagine and serine in the mutant lines than in WT at the maturity stage (**Figures 3D–F**). In comparison to the increase of total N in *Oslht1* lines by about 15-30%, total free amino acids accumulated in the mutants were increased by about 60-120% at the maturation (**Figures 3D, E**). Since amino acids represent the main form of N that was transported over long distances to the reproductive tissues and OsLHT1 is an amino acid transporter (Guo et al., 2020), the results demonstrated that OsLHT1 greatly contributes N re-allocation from source leaves to developing grains.

**Figure 4:**
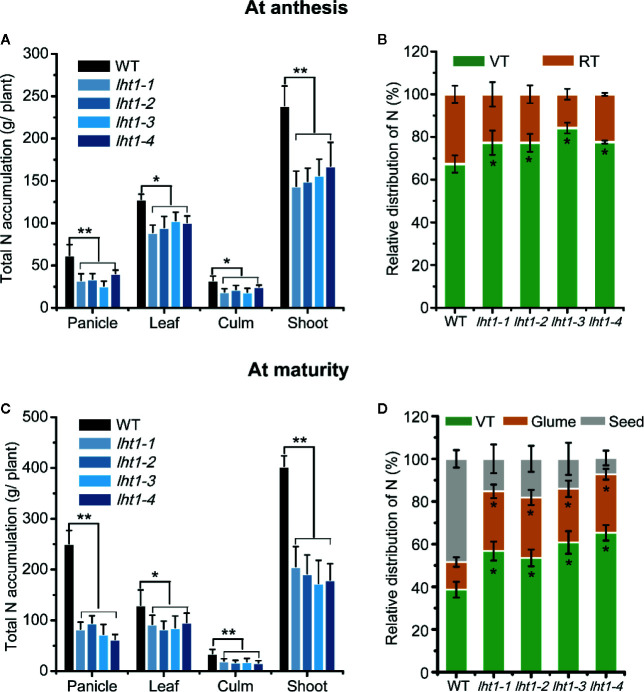
Effect of *OsLHT1* mutation on total N content and relative distribution of N in different organs at anthesis and maturity. Both wild-type (WT, cv. *Nipponbare*) and *Oslht1* mutants were grown in paddy field and sampled at anthesis and mature time. **(A, C)** Total N content in panicles, leaves, culms and total shoot. **(B, D)** Relative amount of N in vegetative tissues (VT) and reproductive tissues (RT). Leaves and culms were considered as vegetative tissues while panicles were considered as reproductive tissues. Values are means ± SD (n = 7). Asterisks indicate significant differences between each *Oslht1* mutant line and wild-type (WT, *Nipponbare*) detected by one-way ANOVA followed by Tukey’s test. *p < 0.05; **p < 0.01.

Since the mutation of *OsLHT1* limited rice growth, as expected, total N per plant was significantly lower in the mutants than in WT at anthesis stage (**Figure 4A**), particularly at maturation stage (**Figure 4C**), while the difference of N in the leaves was relatively smaller than that in other organs (**Figure 4C**). At the anthesis stage, 67% of the total N was found in vegetative tissues and 33% in the reproductive tissues (**Figure 4B**). In contrast, in *Oslht1* mutants, 77% of total N levels remained in the vegetative tissues while about 23% of the total plant N was detected in the reproductive tissues (**Figure 4B**). At the maturity stage, higher percentage of N was allocated to reproductive tissues compared to that at the anthesis stage. *Oslht1* mutants only moved around 40% of total N to the reproductive tissues compared to 62% in WT plants (**Figure 4D**). These data indicated the great contribution of OsLHT1 to leaf-to-panicle allocation in the forms of amino acids.

### Knockout of *OsLHT1* Affected the Components of Grain Storage Compounds and Cooking Texture of Brown Rice

**Figure 5:**
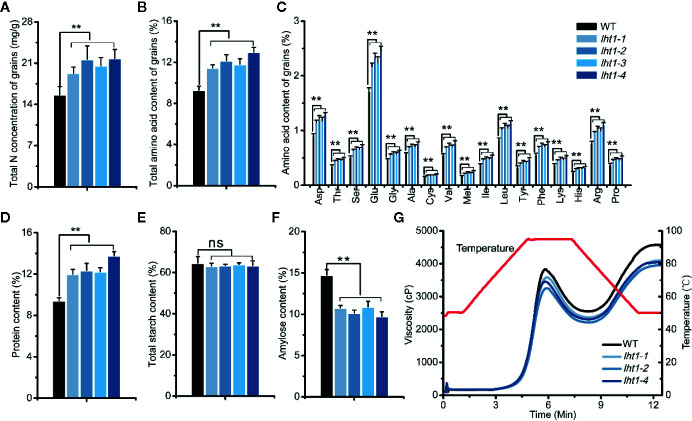
Effect of *OsLHT1* mutation on content of total N, total and individual amino acid, total crude protein, total starch and amylose, and pasting properties of endosperm starch in harvested grains. Both wild-type (WT, cv. *Nipponbare*) and *Oslht1* mutants were grown in paddy field until mature. **(A)** Total N concentration. **(B, C)** Total and individual amino acid content. **(D)** Total crude protein content. **(E)** Total starch content. **(F)** Amylose content. Values in a-f are means ± SD (n = 7). Asterisks indicate significant differences between each *Oslht1* mutant line and WT detected by one-way ANOVA followed by Tukey’s test. **p < 0.01; ns, not significant. **(G)** Pasting properties of endosperm starch. The viscosity value at each temperature is the mean of three replicates. The red line indicates the temperature changes during the measurements.

As reported, the dry seeds of both *Oslht1* mutants and WT plants contained a high amount of total and individual free amino acids in addition to rich starch (**Figure 5**) (Fitzgerald et al., 2009; Li et al., 2018). Compared with WT, *Oslht1* seeds accumulated higher total N and proteins (**Figures 5A, D**), total and individual free amino acids (**Figures 5B, C**) with the increase by up to 30-35%. In contrast, amylose content in *Oslht1* seeds was reduced by about 31% (**Figure 5F**) even though total starch content in the mutant and WT seeds was kept at the same level (**Figure 5E**).

The altered storage compounds in *Oslht1* grains affected cooked texture and pasting properties of the endosperm starch. Both WT and *Oslht1* seed starches showed the similar patterns of pasting with peak viscosity and breakdown when the temperature was increased. However, compared with WT, the peak and cool paste viscosity of the *Oslht1* starch were all decreased (**Figure 5G**).

### Knockout of *OsLHT1* Affected Both N Uptake and Physiological Utilization Efficiency

**Figure 6:**
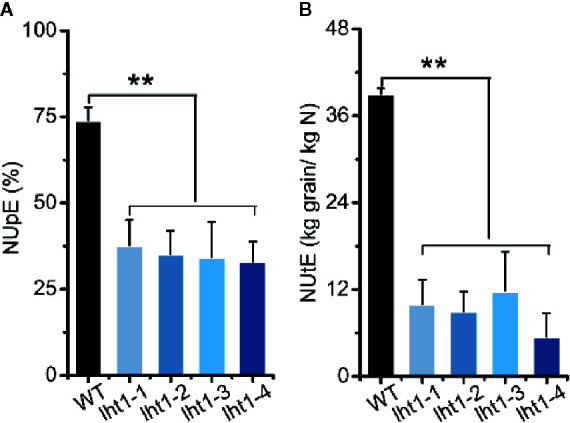
Effect of *OsLHT1* mutation on nitrogen uptake, utilization, and total use efficiency. Both wild-type (WT, cv. *Nipponbare*) and *Oslht1* mutants were grown in paddy field until mature. **(A)** NUpE: nitrogen uptake efficiency = (total accumulated N/total supplied fertilizer N)*100%. **(B)** NUtE: nitrogen utilization efficiency = total grain yield/total accumulated N. Values are means ± SD (n = 7). Asterisks indicate significant differences between each *Oslht1* mutant line and WT detected by one-way ANOVA followed by Tukey’s test. **p < 0.01.

Increasing both the grain and N harvest indexes to drive N acquisition and utilization is an important approach for breeding future high N use efficient cultivars (Xu et al., 2012). In this study, we also analyzed both N uptake efficiency (NUpE) and N utilization efficiency (NUtE) (Xu et al., 2012) of *Oslht1* plants and WT grown in the paddy field with the moderate application level of N fertilizer (180-kg/ha). Knockout of *OsLHT1* resulted in decrease of NUpE by about 50% while it decreased NUtE by about 70% to 90% (**Figure 6**). The extreme low NUtE of *Oslht1* was caused mainly by low grain harvest index (the grain to straw ratio) due to limited N re-allocation.

### Knockout of *OsLHT1* Affected Seed Germination Speed and Rate

**Figure 7:**
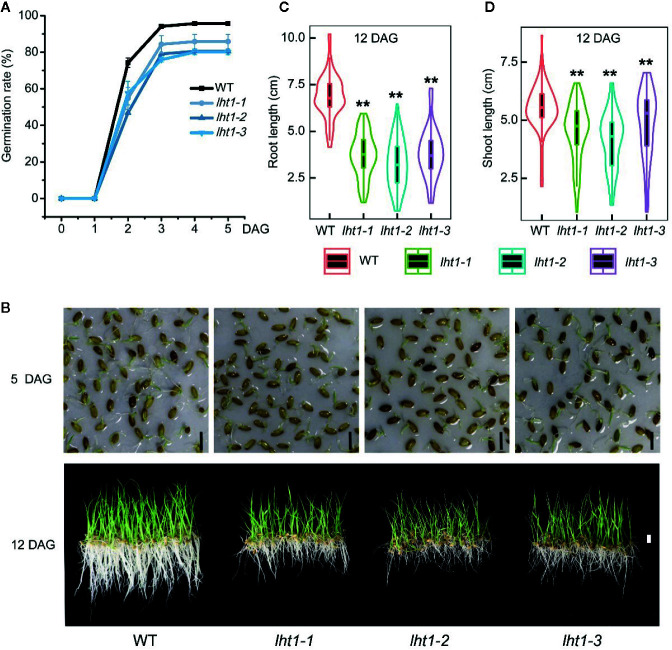
Effect of *OsLHT1* mutation on seed germination, root and shoot growth rate. 100-seeds of each homozygote T4 *Oslht1* mutant line and wild-type (WT, *Nipponbare*) were placed in nutrient-free tap-water. **(A)** Seed germination rate. DAG, day after germination. **(B)** Images of seedlings on nutrient-free water on 5 DAG and 12 DAG. Scale bar= 1 cm. **(C, D)** Root and shoot length of each plant grown in the nutrient-free water for 12 days. Asterisks indicate significant differences between each *Oslht1* mutant line and WT detected by the Student’s t-test, **p < 0.01.

To resolve if the change in *Oslht1* grain storage compounds affects seed viability, we performed germination test of the seeds. The seeds of both WT and mutants began to germinate after soaking in water for 24 h and peaked at 72 h. After 72 h, the germination rate of WT seeds reached 95%, while *Oslht1* seeds germinated only between 80-86% and no further increase after that (**Figure 7A**). Furthermore, the significant differences of seedling growth were also observed between *Oslht1* and WT (**Figure 7B**). After 12-day growth in nutrient-free water, in comparison to WT, *Oslht1* root and shoot length showed incredibly significant decrease (**Figures 7C, D**). The results clearly showed an inhibitory effect on the germination efficacy of the seeds in the loss-of-function of OsLHT1.

## Discussion

### OsLHT1 Functions in Amino Acid Allocation to Panicles and Grains

Amino acids in mature leaves are transported *via* phloem of minor veins to sink tissues (Noctor et al., 2002; Tegeder, 2014). In our previous study, the promoter-GUS assay in rice indicated that OsLHT1 is localized in the leaf blade, especially in the cells of the major and minor veins (Guo et al., 2020). Here we further present high expression of *OsLHT1* in multiple tissues including leaf blades, sheaths, nodes, rachis and spikelets at the anthesis stage, especially in the major and minor veins of leaf blade (**Figure 1**), supporting the role of OsLHT1 in the phloem loading of leaf or root-synthesized amino acids.

Next, OsLHT1 functions in N allocation most probably in the broad spectrum of amino acids as shown in the seedling stage (Guo et al., 2020). In this study, N translocation from source leaves to panicles was clearly established with analysis of four independent *Oslht1* mutant lines. Inactivation of *OsLHT1* resulted in increased accumulation of total N and amino acids in the flag leaves of *Oslht1* mutants from anthesis to maturity stage (**Figure 3**). Considering that OsLHT1 is involved in root-to-shoot allocation and loss function of *OsLHT1* reduced root-synthesized amino acid levels in leaves (Guo et al., 2020), the large amounts of amino acids accumulated in the mutant leaves suggested that the process of amino acids synthesized in source leaves might be stimulated, or less amino acid amounts were allocated into phloem. Since lower percentage of N was contained in the reproductive tissues of *Oslht1* mutants (**Figure 4**), it provides strong support that lack of OsLHT1 function reduced the amino acids allocation from source leaves to panicles. Since OsLHT1 is localized in the major and minor veins of leaf blade and serves as an amino acid importer (**Figure 1F**; Guo et al., 2020), this decrease might result from the reduced amino acid import into phloem. In *Arabidopsis*, many members of AAP family are expressed in the phloem of major and minor veins and contribute to source to sink allocation at the reproductive stage (Sanders et al., 2009; Zhang et al., 2010; Santiago and Tegeder, 2016; Perchlik and Tegeder, 2018). Although AAP and LHT transporters are two distinct groups, they usually have overlapping functions in amino acid translocation (Tegeder and Ward, 2012; Perchlik et al., 2014). Therefore, OsLHT1, a member of LHT family, is one of the major transporters responsible for the transfer process of amino acids in rice.

Amino acids move from the location of the synthesis to the sites of usage to satisfy the demand of plant growth (Tegeder, 2014). At the seedling stage, amino acids are synthesized in the root and exported to supply the developing leaves. In our previous studies, compared with WT, total amounts of free amino acids were reduced in leave blades of *Oslht1* mutants (Guo et al., 2020). At the reproductive stage, amino acids could move from roots to flag leaves and, at the same time, flag leaf could also serve as the source organs to supply amino acids to panicles (Muhammad and Kumazawa, 1974). Since no difference of the free amino acid content in the flag leaf between WT and *Oslht1* lines was observed at the anthesis stage, amino acids imported into flag leaves and exported from flag leaves might be dynamically balanced before anthesis (**Figures 3A, B**). At the maturity stage, much higher percentage of amino acids needs to be allocated to the panicles for meeting the demand of seed production. The higher free leaf amino acids and total N in *Oslht1* mutants than in WT (**Figures 3D–F**) indirectly supports the conclusion that OsLHT1 functions in amino acid allocation to panicles and grains.

### OsLHT1 Contributes to Grain Production and Nutrition Quality and Functionality of Brown Rice

A number of studies indicated that rice sink production is positively related to root N uptake and the amounts of N supplied by vegetative tissues (Fan et al., 2016; Wang et al., 2018). In *Arabidopsis*, LHT1 is crucial for organic N uptake from soil (Ganeteg et al., 2017). *Atlht1* T-DNA insertion mutants displayed dramatic growth inhibition when grown in soil (Hirner et al., 2006). In *Oslht1* mutants, less N distributed into shoot at early stage and panicle sinks at late stage decreased shoot biomass and grain yield production (**Figure 2**; Wang X. et al., 2019; Guo et al., 2020). We have discussed the mechanism of OsLHT1 function in dramatically alteration of rice growth (Guo et al., 2020). We proposed that OsLHT1 mutation could decrease both xylem loading and leaf amino acid import, resulting in N deficiency and growth impairment as observed in *Oslht1* mutant (Hermans et al., 2006; Grechi et al., 2007; Guo et al., 2020). The altered leaf structure and size of *Oslht1* mutants can affect transpiration, water transport and photosynthesis which further limits root N uptake, allocation and re-distribution, as well as downstream carbon-N interaction (Oliveira and Coruzzi, 1999; Lejay et al., 2003; Palenchar et al., 2004; Gutiérrez et al., 2007). In addition, the reduced import of amino acids into mesophyll cells by *Oslht1* mutation could trigger a stress response which in turn restricts plant growth (Pilot et al., 2004; Hirner et al., 2006; Liu et al., 2010; Yang et al., 2014; Sonawala et al., 2018). When an amino acid is not allocated at a sufficient step, it could also serve as the limiting step for rice growth and development (Mo et al., 2006; Muñoz-Bertomeu et al., 2009; Xia et al., 2014). The decreased grain yield was partly due to the extremely low seed setting as well as reduction in grain weight (**Figures 2F, G**). Specific C-, N-regulatory and C/N interaction pathways play crucial roles in seed germination (Osuna et al., 2015). *OsLHT1*-mutation resulted in significant increase of total N and most of individual amino acids, and decrease of amylose concentration in rice grains (**Figure 5**). Internal C/N status could affect seed germination by moderating gibberellin acid and abscisic acid metabolism (Koornneef et al., 2002; Yamaguchi and Kamiya, 2002; Osuna et al., 2015). In addition, seed size is commonly showing positive correlation with seed germination (Ambika et al., 2014). Therefore, the relatively smaller seed size, lower amylose and higher amino acids of *Oslht1* mutants could contribute to the delay of seed germination (**Figure 2G**). Furthermore, decreased grain weight was partially resulted from less seed storage amylose content (**Figure 5F**) while few seed number was caused by decreased amino acid allocation (**Figures 3** and **4**) (Tan et al., 2010; Santiago and Tegeder, 2016). Both root-to-shoot and leaf-to-panicle amino acid allocation were reduced, and eventually led to low grain number (**Figure 2**) (Guo et al., 2020). However, total N and protein levels per grains were still higher in *Oslht1* mutants (**Figures 5A, D**). The results may hint that the trade-off occurs between producing more seed number and keeping high N per seed during the grain filling phase (Seiffert et al., 2004; Drechsler et al., 2015). Overexpressing *AAP1* in pea plants could allocate more N to seeds and produce high seed number (Perchlik and Tegeder, 2017). However, in *Arabidopsis*, decreased amino acid partitioning to sinks led to decreased seed numbers while seed protein levels were unchanged (Schmidt et al., 2007; Santiago and Tegeder, 2016). In rice, amino acid transporter OsAAP6 functions as an important regulator of grain protein content and nutritional quality. OsAAP6 showed no effect on grain yield. However, transgenic plants with higher *OsAAP6* expression levels produced much more grain storage protein (Peng B. et al., 2014). The variation in seed numbers and seed N pools may be caused by the species or other unknown complex mechanism (Fageria and Baligar, 2005; Hirel et al., 2007; Sanders et al., 2009).

The *OsLHT1* mutation induced incredibly low yield and alteration of storage compounds in the kernels. In comparison to WT, *Oslht1* brown rice kernel accumulated significant higher protein and lower amylose (**Figures 5D, F**), which may cause the change of pasting properties of endosperm starch (**Figure 5G**). It has been shown that the formation of protein–starch complexes affects rice starch gelatinization properties, tending to increase flour peak viscosity (Saleh and Meullenet, 2015). The decrease in flour breakdown viscosity of *Oslht1* kernels (**Figure 5G**) suggests a greater protection of the amylose granule integrity. This study demonstrated that changes in rice protein impacted rice flour pasting properties.

Plant N use efficiency (NUE) is inherently complex and it is the combination of N uptake efficiency (NUpE) and N utilization efficiency (NUtE) (Xu et al., 2012). NUE is governed by multiple interacting genetic, environmental and management factors (Balasubramanian et al., 2004; Xu et al., 2012). Many genes involved in N uptake, assimilation, translocation and regulation have been identified to make great contributions to the NUE, such as amino acid transporter AtAAP2 in *Arabidopsis*, nitrate transporter OsNRT1.1b, OsNRT2.3b and ammonium transporter in rice (Zhang et al., 2010; Ranathunge et al., 2014; Bao et al., 2015; Hu et al., 2015; Fan et al., 2016). Rice plants mainly acquire ammonium and transport of N to above ground parts in the forms of amino acids (Xu et al., 2012). Since OsLHT1 plays the key role in the allocation of amino acids from root to shoot (Guo et al., 2020) and N re-distribution from source leaves to developing grains (**Figures 3**–**5**), the inhibitory effect of *Oslht1* mutation on shoot growth acted the feedback role in preventing root N acquisition, which resulted in low NUpE (**Figure 6**). The confined panicle development in *Oslht1* plants decreased grain to straw ratio, which in turn reduced NUtE for root acquired N to produce grains (**Figure 6**).

**Figure 8:**
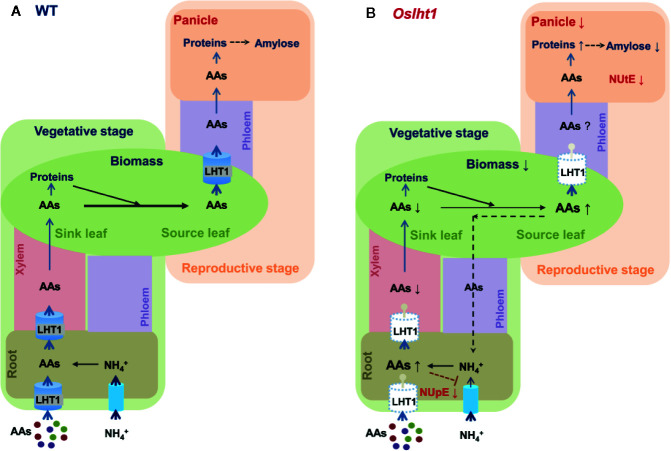
Overview model illustrating the OsLHT1 functions in maintaining both N uptake efficiency and N utilization efficiency in rice. In wild type **(A)**, rice roots directly acquire ammonium and amino acids (AAs) from soil. Ammonium in root cells is rapidly assimilated into AAs that are delivered to shoot (sink leaves) in xylem at vegetative stage. Our previous study (Guo et al., 2020) has shown that OsLHT1 (for simplicity, it is indicated by LHT1 in the figure) directly contributes root acquisition and root to shoot transport of a broad spectrum of amino acids. At reproductive stage, large portion of N is re-allocated mainly in the form of amino acids from source leaves to panicles occurring in the phloem. OsLHT1 plays critical role in this process and functions in grain yield and nutrition quality. **(B)** Knockout of *OsLHT1* dramatically reduces N supply from source leaves to developing panicles which are accompanied by reduced shoot biomass and grain yield with higher storage proteins and lower amylose. The higher levels of N in roots or leaves might negatively affect root ammonium uptake in *oslht1* plants, probably by a feedback regulatory mechanism, which results in lower N uptake efficiency (NUpE). The limited grain yield by loss of *OsLHT1* function results in low N utilization efficiency (NUtE). The sizes of the arrows located to the right of the features analyzed indicate the significant changes in *Oslht1* mutants compared with wild-type plants (up, increase; down, decrease).

In summary, as shown in **Figure 8**, OsLHT1 is not only for root acquisition and long-distance transport of amino acids, it also plays the major role in N allocation from source leaves to developing panicle and grains for reaching the potential grain yield and quality of nutrition and functionality.

## Data Availability Statement

The raw data supporting the conclusions of this article will be made available by the authors, without undue reservation.

## Author Contributions

NG and HQ conceived the research and analyzed the data. NG, JH, and MG performed the experiments. NG, HQ, and GX wrote the article. All authors contributed to the article and approved the submitted version.

## Funding

This work was supported by National Key Research and Development Program of China (2016YFD0100700), Jiangsu Collaborative Innovation Center for Solid Organic Waste Resource Utilization and the Innovative Research Team Development Plan of the Ministry of Education of China (IRT17R56, KYT201802).

## Conflict of Interest

The authors declare that the research was conducted in the absence of any commercial or financial relationships that could be construed as a potential conflict of interest.
